# Photobiomodulation improves frontal lobe cognitive functions and mental health of older adults with non-amnestic mild cognitive impairment: Case studies

**DOI:** 10.3389/fpsyg.2022.1095111

**Published:** 2023-01-10

**Authors:** Mei-Chun Cheung, Tsz-Lok Lee, Sophia L. Sze, Agnes S. Chan

**Affiliations:** ^1^Department of Social Work, The Chinese University of Hong Kong, Shatin, New Territories, Hong Kong SAR, China; ^2^Research Center for Neuropsychological Well-Being, The Chinese University of Hong Kong, Shatin, New Territories, Hong Kong SAR, China; ^3^Neuropsychology Laboratory, Department of Psychology, The Chinese University of Hong Kong, Shatin, New Territories, Hong Kong SAR, China

**Keywords:** photobiomodulation, mild cognitive impairment, frontal lobe cognitive functions, mental health, aging

## Abstract

**Introduction:**

This study investigated the effects of transcranial photobiomodulation (tPBM) on improving the frontal lobe cognitive functions and mental health of older adults.

**Methods:**

Three older adults with mild cognitive impairment (MCI) of the non-amnestic type received 18-session tPBM stimulation for 9 weeks and were assessed with neuropsychological tests of memory and executive functions and standardized questionnaires on depressive and anxiety symptoms, global cognitive functions, and daily functioning abilities before and after tPBM stimulation.

**Results:**

At baseline, their intrusion and/or perseveration errors in a verbal memory test and a fluency test, as measures of the frontal lobe cognitive functions, were in the borderline to severely impaired range at baseline. After tPBM stimulation, the three older adults showed various levels of improvement in their frontal lobe cognitive functions. One older adult’s intrusion and perseveration errors improved from the <1st–2nd percentile (moderately to severely impaired range) to the 41st–69th percentile (average range), another older adult’s intrusion errors improved from the 11th percentile to the 83rd percentile, and the third older adult’s intrusion errors improved from the 5th percentile to the 56th percentile. Moreover, improvements in their anxiety and/or depressive symptoms were also observed. One older adult’s depressive and anxiety symptoms improved from the severe range at baseline to the mild range after the intervention. The other two older adults’ depressive symptoms improved from the mild range at baseline to the normal range after the intervention.

**Discussion:**

These findings provide preliminary support for the potential of tPBM to improve the frontal lobe cognitive functions and mental health of older adults with MCI. Given the small sample size of only three older adults and the absence of a placebo control group, larger randomized controlled studies are needed to confirm its potential.

## 1. Introduction

It has been documented that progressive brain deterioration occurs when we age. A growing body of evidence suggests that the frontal lobe is more susceptible to deterioration than other parts of the brain (West, 1996). Several magnetic resonance imaging (MRI) studies have reported an age-related reduction in gray and white matter volume in various parts of the frontal lobe, such as the dorsolateral prefrontal cortex and the orbitofrontal cortex (Raz et al., 1997, 2005; Kalpouzos et al., 2009). Some diffusion tensor imaging studies have also revealed age-related changes in the white matter microstructure, which are associated with less efficient neural transmission in the prefrontal cortex (Head et al., 2004; Pfefferbaum et al., 2005; Salat et al., 2005). In addition, aging is associated with neurophysiological changes in the frontal lobe. Some positron emission tomography (PET) studies have indicated reduced regional cerebral oxygenation and blood flow in the gray matter of the frontal cortex (Pantano et al., 1984; Bentourkia et al., 2000). In a systematic review of the neuroimaging literature examining cognition in old and young adults, older adults showed more brain activity in the prefrontal regions as compared with young adults across multiple cognitive domains, suggesting that older adults might allocate more neural resources in maintaining behavioral performance at the level seen in young adults (Spreng et al., 2010). Consistent with the associations between aging and neurophysiological changes in the frontal lobe, aging-associated cognitive decline is more prominent in the functions that are primarily mediated by the frontal lobe (West, 1996). Empirical studies have shown that older adults performed poorly in neuropsychological tests of inhibition (West and Alain, 2000; Salthouse, 2010; Fjell et al., 2017), working memory (MacPherson et al., 2002), verbal fluency (Chan and Poon, 1999; Brickman et al., 2005; Van der Elst et al., 2006), problem-solving (Andrés and Van der Linden, 2000), and decision-making (Denburg et al., 2005; Fein et al., 2007; Beitz et al., 2014) as compared to young adults.

The effects of behavior-based interventions on improving the frontal lobe cognitive functions of normal older adults have been examined, such as 4–8 weeks of video game training for improving executive functions (EF), including sustained attention, task switching, and working memory (Basak et al., 2008; Maillot et al., 2012; Anguera et al., 2013). However, only a slight improvement was reported in a meta-analysis study (Karbach and Verhaeghen, 2014). Specifically, it was found that cognitive training that targets improving EF or working memory yielded a mean Cohen’s d of approximately 0.2 in EF/attention measured based on far-transfer tasks. Thus, behavior-based interventions generally appear to produce limited improvements in the frontal lobe cognitive functions of normal older adults. In view of this, there is increasing interest in applying transcranial photobiomodulation (tPBM) as a possible efficient and non-invasive intervention for improving cognitive functions in either normal or clinical populations (for a review, see Chan et al., 2019b; Lee et al., 2022). Previous studies on normal young and older adults have consistently reported that tPBM can significantly improve various aspects of cognitive functions primarily mediated by the frontal lobe, including set-shifting (Blanco et al., 2017a), rule-based category learning (Blanco et al., 2017b), sustained attention (Barrett and Gonzalez-Lima, 2013; Moghadam et al., 2017; Vargas et al., 2017), and working memory (Barrett and Gonzalez-Lima, 2013; Vargas et al., 2017). Specifically, individuals who underwent active tPBM stimulation reacted more quickly in the psychomotor vigilance and parametric go/no-go tasks as compared to the placebo group, suggesting a beneficial effect of tPBM on sustained attention (Barrett and Gonzalez-Lima, 2013; Moghadam et al., 2017). In addition, individuals who completed an active tPBM session showed shorter memory retrieval latency and higher accuracy on the delayed match-to-sample task as compared to the sham control group, suggesting tPBM may improve working memory (Barrett and Gonzalez-Lima, 2013). Furthermore, individuals who had received only a single active tPBM session showed a significant reduction in frontal hemodynamic levels during a difficult task (Chan et al., 2021a) and faster performance in learning category rules (Blanco et al., 2017b) and shifting sets (Blanco et al., 2017a) as compared to individuals who received a sham tPBM. Based on this research evidence, tPBM seems to enhance the cognitive functions mediated by the frontal lobe.

PBM is a non-invasive biological stimulation technique that applies light from the red to near-infrared spectrum (600 to 1,100 nm) to targeted sites of the body (Hamblin, 2016). When PBM is applied on the scalp in an attempt to influence the brain through the skull, this procedure is called tPBM. Because of the relative transparency of biological tissue to PBM light (Jöbsis, 1977; Villringer and Chance, 1997), a small fraction of the delivered light may reach neural tissue (Jagdeo et al., 2012), even though most of the light is absorbed or scattered by the scalp, skull, and cerebrospinal fluid. The underlying mechanisms of PBM are multifaceted and include intracellular activities, extracellular adaptations, and morphological alterations. It has been proposed that the neurobiological effects of PBM occurred primarily at the mitochondrial level. Cytochrome c oxidase (CCO), the terminal enzyme of the mitochondrial respiratory chain, absorbs the photon energy from the tPBM light, resulting in enhanced energy production and oxygen supply to nerve cells and boosted metabolism (Karu, 2000; Wong-Riley et al., 2005; Avci et al., 2013; Hamblin, 2016). It was found that light absorption by CCO leads to the release of nitric oxide (NO), which removes the inhibition on adenosine triphosphate (ATP) production (Sheppard et al., 2005; Lane, 2006; Hamblin, 2008) and provides additional metabolic energy for neural transduction (Tafur and Mills, 2008). These PBM-induced increases in CCO activity (Wong-Riley et al., 2001, 2005; Liang et al., 2008), NO (Sharma et al., 2011), oxygen consumption (Poyton and Ball, 2011), and ATP (Oron et al., 2007; Ying et al., 2008; Dong et al., 2015) have been demonstrated in numerous cell studies. Furthermore, based on the vasodilating effect of NO (Ignarro et al., 1999), studies on both animals (Uozumi et al., 2010) and humans (Salgado et al., 2015) have demonstrated that tPBM increases the diameter of blood vessels, hence increasing regional cerebral blood flow.

Although some studies of tPBM suggest that this intervention seems to have positive effects on the frontal lobe cognitive functions of normal adults and patients with traumatic brain injury (Lee et al., 2022), and depressive and anxiety symptoms in patients with psychological disorders (Gutiérrez-Menéndez et al., 2020), its effect on older adults with mild cognitive impairment (MCI) of the non-amnestic type remains unknown. In this study, we investigated the effect of tPBM on three older adults with non-amnestic MCI who demonstrated impairment in the frontal lobe cognitive functions and depressive and anxiety symptoms at baseline. Given the positive effects of tPBM on improving inhibitory control and mental flexibility (functions mediated by the frontal lobe) of normal older adults in our recent study (Chan et al., 2019a), and the use of tPBM in improving the frontal lobe cognitive functions of patients with traumatic brain injury (TBI) (Naeser et al., 2011, 2014, 2016; Lee et al., 2022) and depressive and anxiety symptoms in clinical patients (Gutiérrez-Menéndez et al., 2020), we anticipated that tPBM stimulation would improve the frontal lobe cognitive functions, and depressive and anxiety symptoms of older adults with non-amnestic MCI. These case studies may provide preliminary support for the potential of tPBM as an intervention for individuals with non-amnestic MCI or cognitive dysfunction associated with the frontal lobe.

## 2. Materials and methods

### 2.1. Participants

Three older adults met the criteria for MCI according to the diagnostic guidelines recommended by the National Institute on Aging and the Alzheimer’s Association (Albert et al., 2011). At baseline, they obtained results above the cutoff level (i.e., 3/5) in the Abbreviated Memory Inventory for Chinese (AMIC) (Lam et al., 2005), which indicated that they had significant subjective complaints of cognitive decline. They reported no history of dementia, other neurological disorders, diabetes, atherosclerosis, hypothyroidism, or cardiac disease. They scored above the clinical cutoff score in the Chinese version of the Mattis Dementia Rating Scale (CDRS) (Chan et al., 2003), suggesting no evidence of dementia. Their functional abilities at baseline were largely independent as measured using the Functional Activities Questionnaire (FAQ) (Pfeffer et al., 1982). They all showed intact memory function (i.e., not meeting the criteria of 1.0 SD below average) as assessed using standardized memory tests, that is, the Hong Kong List Learning Test (HKLLT) (Chan, 2006) and the Rey–Osterrieth Complex Figure Test (Rey-O) (Meyers and Meyers, 1996) at baseline. However, they scored at least 1.0 SD below average in terms of intrusion errors in the HKLLT and the Category Fluency Test (CFT) (Chan and Poon, 1999) and perseveration errors in the CFT. These types of errors are measures of the frontal lobe cognitive functions. Therefore, three older adults were classified as having non-amnestic MCI. This study was approved by the Joint Chinese University of Hong Kong-New Territories East Cluster Clinical Research Ethics Committee (CREC Ref. No.: 2017.354-T). All older adults provided written consent before the study.

### 2.2. Instruments and materials

Before and after tPBM stimulation, each older adult was assessed by well-trained research assistants using standardized neuropsychological tests of memory and executive functions and standardized questionnaires on depressive and anxiety symptoms, global cognitive functions, and daily functioning abilities. Their test results were compared with the norms of their ages and educational levels. The details of the outcome measures are stated below.

The Clinical Dementia Rating Scale (CDR) (Morris, 1993) is a validated semi-structured interview that assesses six cognitive, behavioral, and functional aspects of MCI and dementia. The CDR global score was computed to reflect the severity level of global cognitive deficits.

FAQ (Pfeffer et al., 1982) is a standardized questionnaire that measures 10 aspects of instrumental activities of daily living, which can differentiate MCI from dementia. Examples of daily activities include preparing balanced meals, managing personal finances, and traveling. The total score was computed to indicate the degree of independent living abilities.

HKLLT (Chan, 2006) is a commonly used verbal memory test in Hong Kong that is ecologically validated. In this test, each examinee was asked to learn a list of 16 two-character Chinese words three times and recall them from memory after 30 min. Verbal memory performance was assessed based on the delayed recall score. A higher delayed recall score, suggesting better memory, yielded a higher percentile rank and z-score. The intrusion error of the delayed recall trial was computed as a measure of the frontal lobe cognitive functions. Fewer intrusion errors, indicating better performance, yielded a higher percentile rank and z-score.

Rey-O (Meyers and Meyers, 1996) is a standardized visual memory test that requires the examinee to copy a complex line drawing and then draw it from memory after 30 min. Visual memory performance was assessed based on the delayed recall score. A higher delayed recall score, suggesting better memory, yielded a higher percentile rank and z-score.

CFT (Chan and Poon, 1999) is a test measuring fluency of speech. It requires the examinee to generate as many words as possible that belong to a semantic category (e.g., animals) within a given time interval. The numbers of perseveration and intrusion errors made were adopted as measures of the frontal lobe cognitive functions. Fewer intrusion or perseveration errors, indicating better performance, yield a higher percentile rank and z-score.

The Geriatric Anxiety Scale—10 Item Version (GAS-10) (Mueller et al., 2015) is a self-reported questionnaire measuring 10 aspects of anxiety or stress-related symptoms, such as irritability, tiredness, muscle tension, and restlessness. The total score, ranging from 0 to 30, was computed to reflect the degree of anxiety. Scores of 0–6 are considered normal with minimal anxiety problems, scores of 7–9 suggest a mild level of anxiety, a score of 10 suggests a moderate level of anxiety, and scores at or above 12 suggest a severe level of anxiety.

The Chinese version of the Geriatric Depression Scale—Short Form (CGDS-SF) (Wong et al., 2002) is a standardized self-reported questionnaire measuring 15 aspects of depressive symptoms, such as life satisfaction and a sense of uselessness, helplessness, and sadness. The total score, ranging from 0 to 15, was computed to indicate the degree of depressive symptoms. Scores of 0–4 are considered normal, scores of 5–9 suggest a mild level of depression, and scores of 10 or above suggest a moderate to severe level of depression (Mui, 1996).

### 2.3. tPBM stimulation

The Wisefori 5–3,800 model (Wisefor Ltd. Hong Kong) was used for tPBM stimulation. This device, which is CE-certified and FDA-registered, contains nine individual LED nodes of 1 cm^2^ in size, which were placed on F7, AF7, Fp1, FpZ, Fp2, AF8, F8, Fz, and Cz according to the international 10/10 system. Each LED node emits 810 nm light at an irradiance of 20 mW/cm^2^. The protocol can be changed *via* a smartphone using a patented design. Eighteen tPBM stimulation sessions were implemented for each older adult for 9 weeks (i.e., twice per week). Each stimulation session lasted for around 20 min and consisted of three trials and one-minute breaks between the trials (i.e., first tPBM, 1 min break, second tPBM, 1 min break, third tPBM). The duration of stimulation in each trial was 350 s, which generated a fluence of 7 J/cm^2^. The total surface area of light applied to the skulls was 9 cm^2^. The energy delivered per session was 189 J and the total energy delivered for the 18 sessions was 3,402 J.

## 3. Results

### 3.1. Older adult S1

S1 was a 55-year-old female retired helping professional with 16 years of education. She had a positive history of anxiety and depression. Her level of anxiety symptoms at baseline was assessed to be at the severe level (total score = 15) based on the GAS-10, and her level of depressive symptoms was at the moderate to severe level (total score = 11) as measured by the CGDS-SF (Table 1). Her CGDS-SF score (11/15) was far above the clinical cutoff (8 or above) (Lee et al., 1993), which suggested that her depressive symptoms were clinically significant. At baseline, her age-and education-adjusted CDRS score (total score = 147) was above the clinical cutoff for dementia (Table 1). Her standard CDR global score was 1/3. She reported having moderate impairment in her orientation to time and place and in maintaining life at home and hobbies, mild impairment in memory, and slight problems in managing community affairs. She scored 5/30 in the FAQ (a measure of functional abilities), indicating difficulties in performing some functional activities (Table 1). However, S1 showed intact memory functions at baseline. Her visual memory was assessed to be at the 98th percentile rank (+1.98 SD, clinically classified as in the superior range) based on the Rey-O delayed recall. Her verbal memory was assessed to be in the 72nd percentile (+0.57 SD, clinically classified as in the high average range) based on the HKLLT delayed recall (Table 1).

**Table 1:** Demographics and test scores of each older adult at baseline.

**Participant**	**Age**	**Gender**	**Years of Education**	**CDRS Score**	**CDR Score**	**FAQ Score**	**HKLLT DR (z)**	**Rey-O DR (z)**	**CGDS-SF**	**GAS-10**
S1	55	F	16	147	1	5	+0.57	+1.98	11 (moderate to severe)	15 (severe)
S2	62	F	18	146	0	0	+1.26	−0.95	5 (mild)	4 (minimal)
S3	70	M	13	158	0.5	1	+1.26	−0.38	5 (mild)	3 (minimal)
**Mean**	62.33		15.67	150.33	0.5	2	1.03	0.23	7.00	7.33
**SD**	7.51		2.52	6.66	0.5	2.65	0.40	1.55	3.46	6.66

CDRS, Cantonese version of the Mattis Dementia Rating Scale; CDR, Clinical Dementia Rating Scale; FAQ, Functional Activities Questionnaire; HKLLT DR (*z*), *z*-score of the 30-min delayed recall in the Hong Kong List Learning Test; Rey-O DR (*z*), z-score of the 30-min delayed recall in the Rey–Osterrieth Complex Figure Test; CGDS-SF, Chinese version of Geriatric Depression Scale—Short Form; GAS-10, Geriatric Anxiety Scale—10 Item Version.

**Figure 1:**
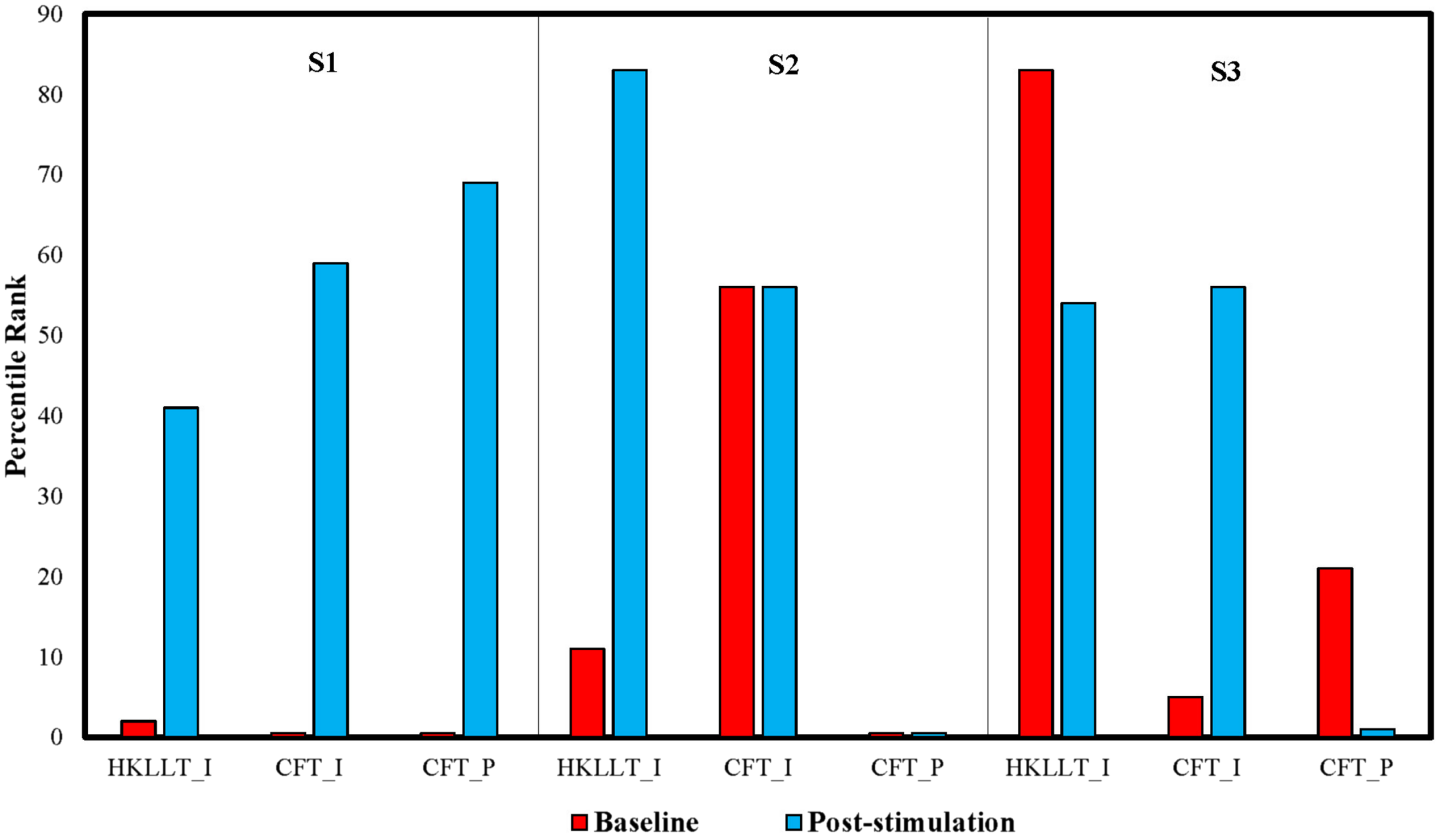
Percentile rank of intrusion errors (I) in the Hong Kong list learning test (HKLLT) and the category fluency test (CFT), and perseveration errors (P) in the CFT at baseline and after tPBM.

She also showed signs of cognitive dysfunction associated with the frontal lobe at baseline, which improved significantly after the intervention. She exhibited substantial intrusion errors in the HKLLT (−2.06 SD; 2nd percentile; clinically classified as in the moderately impaired range) and intrusion and perseveration errors in the CFT (−4.32 SD and − 4.39 SD respectively; <1st percentile; clinically classified as in the severely impaired range). After the intervention, S1’s intrusion and perseveration errors in the CFT improved by 4.55 SD (from the <1st percentile to the 59th percentile) and 4.88 SD (from the <1st percentile to the 69th percentile), respectively. Her intrusion errors in the HKLLT also improved by 1.84 SD (from the 2nd percentile to the 41st percentile), improving from the moderately to severely impaired range to the average range after tPBM stimulation (Figure 1). She also reported better work efficiency after tPBM stimulation.

**Figure 2:**
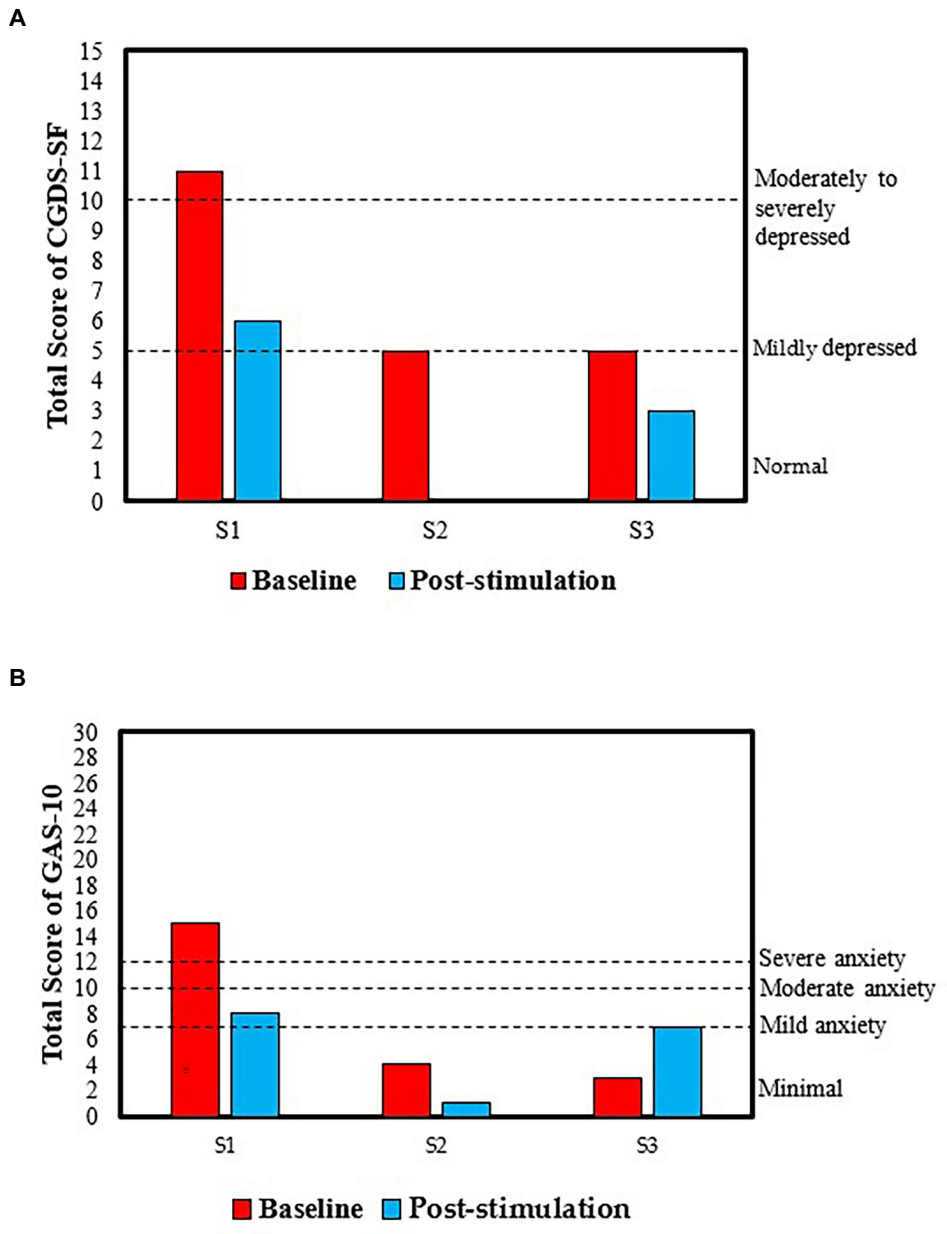
Total scores in **(A)** the Chinese version of the Geriatric Depression Scale—Short Form (CGDS-SF) and **(B)** the Geriatric Anxiety Scale—10 Item Version (GAS-10) at baseline and after tPBM.

Significant improvement was also observed in S1’s depressive and anxiety symptoms. According to her CGDS-SF scores, she reported a moderate to severe level of depressive symptoms (total score = 11) at baseline but a mildly depressed level (total score = 6) after the intervention (Figure 2A). In addition, she reported a severe level of anxiety symptoms (total score = 15) at baseline but a mild level (total score = 8) after tPBM as measured based on the GAS-10 (Figure 2B). Her improved self-rated anxiety symptoms were in line with her reported subjective feeling of reduced nervousness and compulsive acts, e.g., repeated checking behavior, after the intervention.

Moreover, her CDR global score dropped from 1 to 0 (Table 2). She initially reported mild to moderate impairments in orientation, life at home, hobbies, and memory at baseline, which were no longer a problem after tPBM. Her score in the FAQ dropped slightly from 5 to 4 (Table 2), which remained at the below-cutoff level, and she still had difficulties in performing some functional activities. In addition, her visual and verbal memory remained in the superior (99^th^ percentile) and the high average (81st percentile) ranges, respectively, after the intervention (Figure 3).

**Table 2:** Scores obtained in cognitive and functional abilities tests before and after tPBM.

**Measures**	**S1**		**S2**		**S3**	
	**Before**	**After**	**Before**	**After**	**Before**	**After**
CDR^^^	1	0	0	0	0.5	0.5
FAQ^^^	5	4	0	0	1	0
HKLLT_I (z)^#^	−2.06	−0.22	−1.23	+0.94	–	–
CFT_I (z)^#^	−4.32	+0.23	–	–	−1.60	+0.27
CFT_P (z)^#^	−4.39	+0.49	−3.18	−3.18	–	–

CDR, Clinical Dementia Rating Scale; FAQ, Functional Activities Questionnaire; HKLLT_I (z), intrusion errors in the Hong Kong List Learning Test in terms of z-scores; CFT_I (*z*), intrusion errors in the Category Fluency Test (CFT) in terms of z-scores; CFT_P (*z*), perseveration errors in the Category Fluency Test in terms of *z*-scores.

^ Higher scores indicate more significant problems/difficulties.

# Performance scores at least 1.0 SD below average at baseline in intrusion and perseveration errors are presented. Positive changes after tPBM indicate better performance.

**Figure 3:**
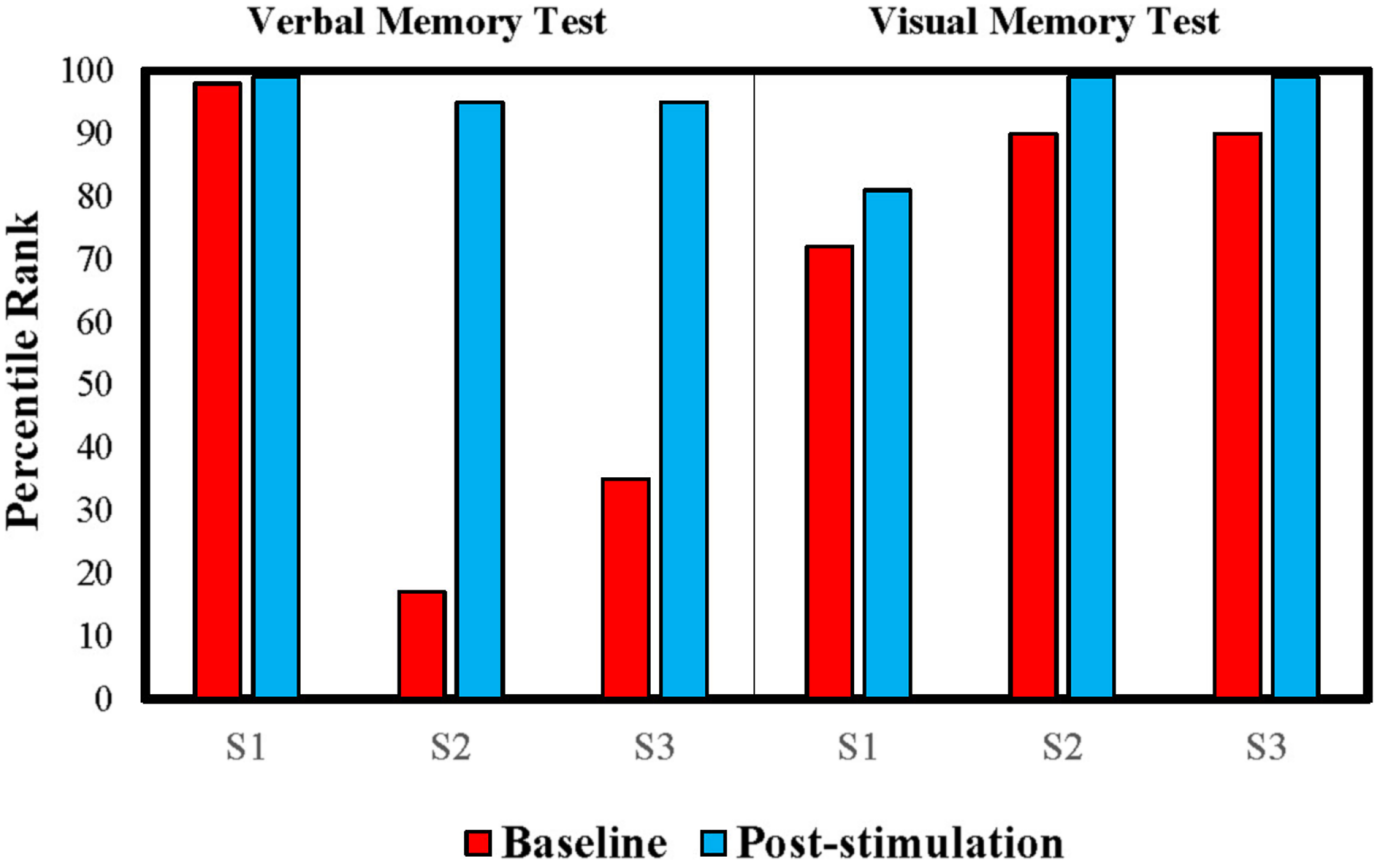
Percentile rank of each older adult in visual and verbal memory tests before and after tPBM. Older adults’ visual memory was measured based on the 30-min delayed recall in the Rey–Osterrieth Complex Figure Test (Rey-O), whereas their verbal memory was measured based on the 30-min delayed recall in the HKLLT.

### 3.2. Older adult S2

S2 was a 62-year-old female with 18 years of education who was a retired business manager. At baseline, her age-and education-adjusted CDRS score (total score = 146) was above the clinical cut-off, which suggested that her cognitive function was not consistent with dementia. She scored zero in both the CDR and FAQ (Table 1). Her memory function was also within normal limits, and her verbal memory as measured based on the HKLLT (+1.26 SD; 90th percentile; high average range) was better than her visual memory (−0.95 SD; 17th percentile; low average range) as measured based on the Rey-O (Table 1). Moreover, her reported depressive symptoms were mild as measured based on her CGDS-SF total score (5/15), and her anxiety symptoms were minimal as measured based on her GAS-10 total score (4/30) (Table 1).

Similar to older adult S1, S2 showed signs of cognitive dysfunction associated with the frontal lobe at baseline. Her intrusion errors in the HKLLT were at the 11th percentile level (−1.23 SD; borderline range), and her perseveration errors in the CFT were at the <1st percentile level (−3.18 SD; severely impaired range). After the intervention, her intrusion errors in the HKLLT improved by 2.17 SD from the borderline range (11th percentile) to the high average range (83rd percentile) (Figure 1). Her intrusion errors in the CFT remained at the 56th percentile level (+0.16 SD; average range). However, her perseveration errors in the CFT remained at the <1st percentile level after the intervention. According to her feedback, she experienced some improvements in her memory and became more fluent and experienced less difficulty when giving a speech during the course of the intervention.

S2 also showed some improvement in her level of depressive and anxiety symptoms. At baseline, her depressive symptoms as measured based on the CGDS-SF improved from a mild level (total score = 5) to a normal level (total score = 0) (Figure 2A). Furthermore, her initially minimal level of anxiety symptoms, as measured based on the GAS-10 score, had further decreased with the score changing from 4 to 1 (Figure 2B).

According to her scores in the CDR and FAQ, she continued to have intact global cognitive and functional abilities in the pre-and post-intervention assessments (Table 2). Interestingly, her intact memory function at baseline was further improved after the intervention (Figure 3). Her visual memory improved by 2.64 SD from the low average range (17th percentile) to the superior range (95th percentile) in the Rey-O, and her verbal memory improved by 1.19 SD from the high average range (90th percentile) to the superior range (99th percentile) in the HKLLT.

### 3.3. Older adult S3

S3 was a 70-year-old male retired land surveyor with 13 years of education. At baseline, his adjusted CDRS score (total score = 158) was above the clinical cut-off. His scores in the CDR and FAQ were 0.5/3 and 1/30, respectively, suggesting that his global cognitive functions and functional abilities were generally intact (Table 1). In addition, he had intact memory function at baseline. His visual memory was at the 35th percentile level (−0.38 SD; average range) and his verbal memory was at the 90th percentile level (+1.26 SD; high average range) (Table 1). His depressive symptoms as reported in the CGDS-SF were at a mild level (5/15) and his anxiety symptoms in the GAS-10 were also at a minimal level (3/30) (Table 1).

At baseline, S3’s intrusion errors in the CFT were at the 5th percentile level (−1.6 SD; mildly impaired range). After the intervention, his intrusion errors improved by 1.87 SD from the 5th percentile to the 56th percentile (from the mildly impaired range to the average range) (Figure 1). This indicates that his inhibitory control had largely improved. His intrusion errors in the HKLLT remained within normal limits after the intervention, with the percentile rank slightly decreasing from the 83rd percentile (high average range) to the 54th percentile (average range). However, he made more perseveration errors in the CFT after the intervention. His percentile rank dropped from the 21st percentile (low average range) at baseline to the 1st percentile (moderately impaired range) after the intervention.

S3’s reported depressive symptoms, as measured using the CGDS-SF, were slightly reduced from mild level (total score = 5) to normal level (total score = 3), suggesting some improvement in his depressive symptoms (Figure 2A). However, his anxiety symptoms increased from a minimal level (total score = 3) to a mild level (total score = 7), according to his GAS-10 score (Figure 2B).

S3’s CDR global score remained at 0.5 (Table 2), and his CDR-Memory score dropped from moderate impairment (score = 2) to mild impairment (score = 1). Similar to S2, he also showed some improvement in his memory as measured by the HKLLT and the Rey-O (Figure 3). His verbal memory improved by 0.98 SD from the 90th percentile (high average range) to the 99th percentile (superior range), and his visual memory improved by 0.8 SD from the 35th percentile (average range) to the 95th percentile (superior range). In the FAQ, his total score improved from 1 to 0 (Table 2). He had difficulty remembering appointments, family occasions, holidays, and medications at baseline; however, he was able to remember these events independently after tPBM.

## 4. Discussion

This study showed that three older adults with non-amnestic MCI, who received tPBM stimulation over 9 weeks, showed different levels of reduction in intrusion and perseveration errors (indices of inhibitory control and mental flexibility as mediated by the frontal lobe). Specifically, S1 showed improvements in all three outcome measures, S2 showed reduced intrusion errors in the HKLLT, and S3 showed reduced intrusion errors in the CFT. The stimulation was well-tolerated by all older adults, and no side effects or adverse events were reported. The extent of intrusion/perseveration improvement could be as large as an increase of 4.88 SD, with the mean change over three older adults being an increase of 2.55 SD. All three older adults demonstrated a reduction in depressive symptoms, and two older adults (S1 and S2) showed reduced anxiety symptoms after tPBM stimulation. Similar to our previous case reports on amnestic MCI (Chan et al., 2021b), two older adults (S2 and S3) with non-amnestic MCI in the present study also showed further enhancements in visual and verbal memory of between +0.8 SD and + 2.64 SD, despite their within-normal-range memory function at baseline. The other older adult (S1), who displayed the high average to superior range of memory performance at baseline, remained at this high level after the intervention. Given the lack of a control group, one may argue whether the improvements were due to the intervention, or other factors, such as repeated testing or the passing of time. As reported in our previous case reports (Chan et al., 2021b), three amnestic MCI older adults who did not receive tPBM failed to show a positive change in their re-assessment after 9 weeks. Their mean change in memory measures was −0.23 SD, thus indicating a deterioration in performance. Therefore, practice effects are likely not the reason for the three older adults with non-amnestic MCI who showed various degrees of cognitive improvement in the present study despite a mixed pattern of changes. In addition, since three older adults had received a diagnosis of MCI before tPBM stimulation, any natural spontaneous recovery of impairment in cognitive function within 3 months is also quite unlikely. Still, given the small sample size of only three older adults and the absence of a placebo control group, these findings can only suggest the potential of tPBM to improve non-amnestic MCI conditions. Larger randomized placebo-controlled studies are needed to confirm its potential.

The improved frontal lobe cognitive functions reported after tPBM stimulation in these case reports are consistent with the findings of some previous studies on MCI (Salehpour et al., 2019), TBI (Naeser et al., 2011, 2014, 2016), older adults with subjective memory complaints (Vargas et al., 2017), and normal individuals (Blanco et al., 2017a; Chan et al., 2019a). In particular, Salehpour et al. (2019) reported significantly improved test performance in the executive functions and working memory of an individual with MCI after twice-daily transcranial and intranasal PBM therapy for 4 weeks. Two studies conducted by Naeser et al. (2011, 2014) also demonstrated improved inhibitory control in the Stroop test after tPBM in patients with chronic TBI. Our previous study (Chan et al., 2019a) on normal older adults showed similar improvements in inhibitory control and mental flexibility after a single tPBM stimulation. Nevertheless, empirical studies on the effects of tPBM on the frontal lobe cognitive functions and its underlying neural mechanism are relatively limited due to a small sample size. Therefore, it is worthwhile to recruit more older adults with non-amnestic MCI or cognitive dysfunction associated with the frontal lobe to investigate this issue in future studies.

Similar to our previous study on older adults with amnestic MCI (Chan et al., 2021b), improved depressive and anxiety symptoms were reported in three older adults with non-amnestic MCI after the intervention. Particularly, in the case of the older adult S1, her reported depressive symptoms improved from the moderate-to-severe level (total score = 11) to the mild level (total score = 6) as measured by the CGDS-SF. Such a reduction in this score signifies an improvement from a clinically significant level (i.e., above the clinical cutoff) to a non-significant level (i.e., below the clinical cutoff). Her anxiety symptoms also improved from a severe level (total score = 15) to a mild level (total score = 8) in the GAS-10. Furthermore, she felt less nervous and compulsive in daily life. Older adult S2 also showed an improvement in depressive and anxiety symptoms after tPBM. Her mild level of depressive symptoms (total score = 5) in the CGDS-SF at baseline returned to a normal level (total score = 0) after tPBM. In addition, her initial minimal level of anxiety symptoms was further reduced, and her GAS-10 total score decreased from 4 to 1. Older adult S3 also showed a slight improvement in his depressive symptoms from the mildly depressed level (total score = 5) to the normal level (total score = 3). However, he demonstrated a mild increase in his anxiety symptoms after the intervention for an unknown reason. Recent systematic reviews (Caldieraro and Cassano, 2019; Gutiérrez-Menéndez et al., 2020) suggested that tPBM may be a possible effective treatment for psychological disorders. Caldieraro and Cassano (2019) indicated that tPBM induced significant antidepressant and anxiolytic effects and showed good tolerability among patients with major depressive disorder and anxiety disorders in the clinical studies. Nevertheless, given the heterogeneity of the population and treatment parameters, and the lack of appropriate control in previous studies, the actual effect of tPBM on depressive and anxiety symptoms remains uncertain and further investigation is needed.

A recent study recruited 32 male and 24 female patients with dementia to investigate the gender differences for the improvement after tPBM stimulation (Qi et al., 2021). They did not find statistically significant gender-based differences in terms of the changes in the neuropsychological test performance after tPBM. Although the present study has an insufficient sample size to conclude on the gender effects, our positive outcomes with two female and one male older adults with non-amnestic MCI generally suggest that tPBM can benefit both female and male older adults. To determine whether the effects of tPBM on older adults with non-amnestic MCI are gender-neutral, a power analysis is conducted to estimate the required sample size. Using the three primary outcome measures (i.e., intrusion errors in the HKLLT, and intrusion and perseveration errors in the CFT, expressed by *z*-scores), the average effect size of the change is 0.62 (range = 0.28–0.94). As this study yields an average statistical power of 0.11 (range = 0.06–0.16), it is estimated that around 22 non-amnestic MCI older adults will be required to achieve a power of 0.8 at the 0.05 alpha level. Therefore, future studies are needed to recruit more older adults to substantiate the effects of gender in response to tPBM. Finally, there is still no consensus on the optimal treatment protocols for the clinical application of tPBM in different clinical populations, and the dosages and exposure times vary across studies (Lee et al., 2022). For instance, improvements in cognitive functions, improved sleep and mood, reduction in anxiety, and positive changes in daily routine were demonstrated after two 6-min sessions daily with a power density of 23.1 mW/cm^2^ for 8 weeks (Nizamutdinov et al., 2021). Therefore, it is likely that the improvement in older adults with non-amnestic MCI might be even higher if they receive more exposure to tPBM stimulation at a higher dosage. Further research may consider evaluating the treatment effects in response to different dosages and exposure times.

## 5. Conclusion

The current study has provided preliminary empirical evidence for the positive effects of an 18-session tPBM stimulation in improving the frontal lobe cognitive functions and depressive and anxiety symptoms of three older adults with non-amnestic MCI. At baseline, their intrusion and/or perseveration errors in a verbal memory test and a fluency test, as measures of the frontal lobe cognitive functions, were in the borderline to severely impaired range. After tPBM, they demonstrated various levels of improvement in their frontal lobe cognitive functions, and some functions even fell into the average range. Improvements in depressive/anxiety symptoms were also observed after the intervention. These findings provide preliminary support for tPBM as a non-invasive intervention to improve the frontal lobe cognitive functions and mental health of older adults with MCI. Further investigation through larger randomized placebo-controlled studies is needed to confirm its potential.

## Data availability statement

The raw data supporting the conclusions of this article will be made available by the authors, without undue reservation.

## Ethics statement

The studies involving human participants were reviewed and approved by the Joint Chinese University of Hong Kong – New Territories East Cluster Clinical Research Ethics Committee (CREC Ref. No.: 2017.354-T, date of approval: 20 October 2017). The patients/participants provided their written informed consent to participate in this study.

## Author contributions

M-CC and AC: conceptualization. T-LL, SS, and AC: methodology and writing—original draft preparation. T-LL and SS: formal analysis. M-CC, T-LL, SS, and AC: writing—review and editing. AC: supervision and funding acquisition. All authors contributed to the article and approved the submitted version.

## Funding

This research was funded by a grant from the Research Grant Council of the Hong Kong Special Administrative Region, China, to The Chinese University of Hong Kong, grant number: CUHK 14606519 (AC).

## Conflict of interest

The authors declare that the research was conducted in the absence of any commercial or financial relationships that could be construed as a potential conflict of interest.

## Publisher’s note

All claims expressed in this article are solely those of the authors and do not necessarily represent those of their affiliated organizations, or those of the publisher, the editors and the reviewers. Any product that may be evaluated in this article, or claim that may be made by its manufacturer, is not guaranteed or endorsed by the publisher.

## References

[ref1] Albert M. S. DeKosky S. T. Dickson D. Dubois B. Feldman H. H. Fox N. C.. (2011). The diagnosis of mild cognitive impairment due to Alzheimer’s disease: recommendations from the National Institute on Aging-Alzheimer’s association workgroups on diagnostic guidelines for Alzheimer’s disease. Alzheimers Dement. 7, 270–279. doi: 10.1016/j.jalz.2011.03.008, PMID: 21514249 PMC3312027

[ref2] Andrés P. Van der Linden M. (2000). Age-related differences in supervisory attentional system functions. J. Gerontol. B Psychol. Sci. Soc. Sci. 55, P373–P380. doi: 10.1093/geronb/55.6.P373, PMID: 11078107

[ref3] Anguera J. A. Boccanfuso J. Rintoul J. L. Al-Hashimi O. Faraji F. Janowich J.. (2013). Video game training enhances cognitive control in older adults. Nature 501, 97–101. doi: 10.1038/nature12486, PMID: 24005416 PMC3983066

[ref4] Avci P. Gupta A. Sadasivam M. Vecchio D. Pam Z. Pam N.. (2013). Low-level laser (light) therapy (LLLT) in skin: stimulating, healing, restoring. Semin. Cutan. Med. Surg. 32, 41–52. 24049929 PMC4126803

[ref5] Barrett D. Gonzalez-Lima F. (2013). Transcranial infrared laser stimulation produces beneficial cognitive and emotional effects in humans. Neuroscience 230, 13–23. doi: 10.1016/j.neuroscience.2012.11.016, PMID: 23200785

[ref6] Basak C. Boot W. R. Voss M. W. Kramer A. F. (2008). Can training in a real-time strategy video game attenuate cognitive decline in older adults? Psychol. Aging 23, 765–777. doi: 10.1037/a0013494, PMID: 19140648 PMC4041116

[ref7] Beitz K. M. Salthouse T. A. Davis H. P. (2014). Performance on the Iowa gambling task: from 5 to 89 years of age. J. Exp. Psychol. 143, 1677–1689. doi: 10.1037/a0035823, PMID: 24512562 PMC4115037

[ref8] Bentourkia M. Bol A. Ivanoiu A. Labar D. Sibomana M. Coppens A.. (2000). Comparison of regional cerebral blood flow and glucose metabolism in the normal brain: effect of aging. J. Neurol. Sci. 181, 19–28. doi: 10.1016/S0022-510X(00)00396-8, PMID: 11099707

[ref9] Blanco N. J. Maddox W. T. Gonzalez-Lima F. (2017a). Improving executive function using transcranial infrared laser stimulation. J. Neuropsychol. 11, 14–25. doi: 10.1111/jnp.12074, PMID: 26017772 PMC4662930

[ref10] Blanco N. J. Saucedo C. L. Gonzalez-Lima F. (2017b). Transcranial infrared laser stimulation improves rule-based, but not information-integration, category learning in humans. Neurobiol. Learn. Mem. 139, 69–75. doi: 10.1016/j.nlm.2016.12.016, PMID: 28039085

[ref11] Brickman A. M. Paul R. H. Cohen R. A. Williams L. M. MacGregor K. L. Jefferson A. L.. (2005). Category and letter verbal fluency across the adult lifespan: relationship to EEG theta power. Arch. Clin. Neuropsychol. 20, 561–573. doi: 10.1016/j.acn.2004.12.006, PMID: 15939182 PMC2758771

[ref12] Caldieraro M. A. Cassano P. (2019). Transcranial and systemic photobiomodulation for major depressive disorders: a systematic review of efficiency, tolerability and biological mechanisms. J. Affect. Disord. 243, 262–273. doi: 10.1016/j.jad.2018.09.048, PMID: 30248638

[ref13] Chan A. S. (2006). Hong Kong List Learning Test. 2nd Edn. Hong Kong SAR, China: Department of Psychology, The Chinese University of Hong Kong.

[ref14] Chan A. S. Choi A. Chiu H. Lam L. (2003). Clinical validity of the Chinese version of Mattis dementia rating scale in differentiating dementia of Alzheimer’s type in Hong Kong. J. Int. Neuropsychol. Soc. 9, 45–55. doi: 10.1017/S1355617703910058, PMID: 12570357

[ref15] Chan A. S. Lee T. L. Hamblin M. R. Cheung M. C. (2021a). Photobiomodulation enhances memory processing in older adults with mild cognitive impairment: a functional near-infrared spectroscopy study. J. Alzheimers Dis. 83, 1471–1480. doi: 10.3233/JAD-201600, PMID: 33998541

[ref16] Chan A. S. Lee T. L. Sze S. L. Hamblin M. R. (2021b). Photobiomodulation improves memory in mild cognitive impairment: three case reports. Alzheimers Dis. Dement. 5, 126–131. doi: 10.36959/734/383

[ref17] Chan A. S. Lee T. L. Yeung M. K. Hamblin M. R. (2019a). Photobiomodulation improves the frontal cognitive function of older adults. Int. J. Geriatr. Psychiatry 34, 369–377. doi: 10.1002/gps.5039, PMID: 30474306 PMC6333495

[ref18] Chan A. S. Poon M. W. (1999). Performance of 7-to 95-year-old individuals in a Chinese version of the category fluency test. J. Int. Neuropsychol. Soc. 5, 525–533. doi: 10.1017/S135561779956606X, PMID: 10561933

[ref19] Chan A. S. Yeung M. K. Lee T. L. (2019b). “Can photobiomodulation enhance brain function in older adults?” in Photobiomodulation in the Brain: Low-level Laser (light) Therapy in Neurology and Neuroscience. eds. Hamblin M. Huang Y. Y. (New York, NY: Academic Press), 427–446.

[ref20] Denburg N. L. Tranel D. Bechara A. (2005). The ability to decide advantageously declines prematurely in some normal older persons. Neuropsychologia 43, 1099–1106. doi: 10.1016/j.neuropsychologia.2004.09.012, PMID: 15769495

[ref21] Dong T. Zhang Q. Hamblin M. R. Wu M. X. (2015). Low-level light in combination with metabolic modulators for effective therapy of injured brain. J. Cereb. Blood Flow Metab. 35, 1435–1444. doi: 10.1038/jcbfm.2015.87, PMID: 25966949 PMC4640344

[ref22] Fein G. McGillivray S. Finn P. (2007). Older adults make less advantageous decisions than younger adults: cognitive and psychological correlates. J. Int. Neuropsychol. Soc. 13, 480–489. doi: 10.1017/S135561770707052X, PMID: 17445297 PMC1868494

[ref23] Fjell A. M. Sneve M. H. Grydeland H. Storsve A. B. Walhovd K. B. (2017). The disconnected brain and executive function decline in aging. Cereb. Cortex 27, 2303–2317. doi: 10.1093/cercor/bhw082, PMID: 27073220

[ref24] Gutiérrez-Menéndez A. Marcos-Nistal M. Méndez M. Arias J. L. (2020). Photobiomodulation as a promising new tool in the management of psychological disorders: a systematic review. Neurosci. Biobehav. Rev. 119, 242–254. doi: 10.1016/j.neubiorev.2020.10.002, PMID: 33069687

[ref25] Hamblin M. R. (2008). The role of nitric oxide in low level light therapy. Paper presented at the SPIE BiOS, San Jose, CA, USA, February 25, 2008.

[ref26] Hamblin M. R. (2016). Shining light on the head: Photobiomodulation for brain disorders. BBA Clin. 6, 113–124. doi: 10.1016/j.bbacli.2016.09.002, PMID: 27752476 PMC5066074

[ref27] Head D. Buckner R. L. Shimony J. S. Williams L. E. Akbudak E. Conturo T. E.. (2004). Differential vulnerability of anterior white matter in nondemented aging with minimal acceleration in dementia of the Alzheimer type: evidence from diffusion tensor imaging. Cereb. Cortex 14, 410–423. doi: 10.1093/cercor/bhh003, PMID: 15028645

[ref28] Ignarro L. J. Cirino G. Casini A. Napoli C. (1999). Nitric oxide as a signaling molecule in the vascular system: an overview. J. Cardiovasc. Pharmacol. 34, 879–886. doi: 10.1097/00005344-199912000-00016, PMID: 10598133

[ref29] Jagdeo J. R. Adams L. E. Brody N. I. Siegel D. M. (2012). Transcranial red and near infrared light transmission in a cadaveric model. PLoS One 7:e47460. doi: 10.1371/journal.pone.0047460, PMID: 23077622 PMC3471828

[ref30] Jöbsis F. F. (1977). Noninvasive, infrared monitoring of cerebral and myocardial oxygen sufficiency and circulatory parameters. Science 198, 1264–1267. doi: 10.1126/science.929199, PMID: 929199

[ref31] Kalpouzos G. Chetelat G. Baron J. C. Landeau B. Mevel K. Godeau C.. (2009). Voxel-based mapping of brain gray matter volume and glucose metabolism profiles in normal aging. Neurobiol. Aging 30, 112–124. doi: 10.1016/j.neurobiolaging.2007.05.019, PMID: 17630048

[ref32] Karbach J. Verhaeghen P. (2014). Making working memory work: a meta-analysis of executive-control and working memory training in older adults. Psychol. Sci. 25, 2027–2037. doi: 10.1177/0956797614548725, PMID: 25298292 PMC4381540

[ref33] Karu T. (2000). Mechanisms of low-power laser light action on cellular level. Paper presented at the EOS/SPIE European Bio-medical Optics Week, Amsterdam, Netherlands, November 3, 2000.

[ref34] Lam L. C. Lui V. W. Tam C. W. Chiu H. F. (2005). Subjective memory complaints in Chinese subjects with mild cognitive impairment and early Alzheimer's disease. Int. J. Geriatr. Psychiatry 20, 876–882. doi: 10.1002/gps.1370, PMID: 16116581

[ref35] Lane N. (2006). Cell biology: power games. Nature 443, 901–903. doi: 10.1038/443901a 17066004

[ref36] Lee H. C. B. Chiu H. F. K. Kwok W. Y. Leung C. M. Kwong P. K. Chung D. W. S.. (1993). Chinese elderly and the GDS short form: a preliminary study. Clin. Gerontol. 14, 37–42.

[ref37] Lee T. L. Ding Z. Chan A. S. (2022). Can transcranial photobiomodulation improve cognitive function? A systematic review of human studies. Ageing Res. Rev. 83:101786. doi: 10.1016/j.arr.2022.101786, PMID: 36371017

[ref38] Liang H. L. Whelan H. T. Eells J. T. Wong-Riley M. T. (2008). Near-infrared light via light-emitting diode treatment is therapeutic against rotenone and 1-methyl-4-phenylpyridinium ion-induced neurotoxicity. Neuroscience 153, 963–974. doi: 10.1016/j.neuroscience.2008.03.042, PMID: 18440709 PMC2587428

[ref39] MacPherson S. E. Phillips L. H. Della Sala S. (2002). Age, executive function and social decision making: a dorsolateral prefrontal theory of cognitive aging. Psychol. Aging 17, 598–609. doi: 10.1037/0882-7974.17.4.598, PMID: 12507357

[ref40] Maillot P. Perrot A. Hartley A. (2012). Effects of interactive physical-activity video-game training on physical and cognitive function in older adults. Psychol. Aging 27, 589–600. doi: 10.1037/a0026268, PMID: 22122605

[ref41] Meyers J. Meyers K. (1996). Rey Complex Figure and the Recognition Trial: Professional Manual. Supplemental Norms for Children and Adolescents. Odessa, FL, USA: Psychological Assessment Resources.

[ref42] Moghadam H. S. Nazari M. A. Jahan A. Mahmoudi J. Salimi M. M. (2017). Beneficial effects of transcranial light emitting diode (LED) therapy on attentional performance: an experimental design. Iran Red Crescent Med J 19:e44513. doi: 10.5812/ircmj.44513

[ref43] Morris J. C. (1993). The clinical dementia rating (CDR): current version and scoring rules. Neurology 43, 2412–2414. doi: 10.1212/WNL.43.11.2412-a, PMID: 8232972

[ref44] Mueller A. E. Segal D. L. Gavett B. Marty M. A. Yochim B. June A.. (2015). Geriatric anxiety scale: item response theory analysis, differential item functioning, and creation of a ten-item short form (GAS-10). Int. Psychogeriatr. 27, 1099–1111. doi: 10.1017/S1041610214000210, PMID: 24576589

[ref45] Mui A. C. (1996). Geriatric depression scale as a community screening instrument for elderly Chinese immigrants. Int. Psychogeriatr. 8, 445–458. doi: 10.1017/S1041610296002803, PMID: 9116180

[ref46] Naeser M. A. Martin P. I. Ho M. D. Krengel M. H. Bogdanova Y. Knight J. A.. (2016). Transcranial, red/near-infrared light-emitting diode therapy to improve cognition in chronic traumatic brain injury. Photomed. Laser Surg. 34, 610–626. doi: 10.1089/pho.2015.4037, PMID: 28001756

[ref47] Naeser M. A. Saltmarche A. Krengel M. H. Hamblin M. R. Knight J. A. (2011). Improved cognitive function after transcranial, light-emitting diode treatments in chronic, traumatic brain injury: two case reports. Photomed. Laser Surg. 29, 351–358. doi: 10.1089/pho.2010.2814, PMID: 21182447 PMC3104287

[ref48] Naeser M. A. Zafonte R. Krengel M. H. Martin P. I. Frazier J. Hamblin M. R.. (2014). Significant improvements in cognitive performance post-transcranial, red/near-infrared light-emitting diode treatments in chronic, mild traumatic brain injury: open-protocol study. J. Neurotrauma 31, 1008–1017. doi: 10.1089/neu.2013.3244, PMID: 24568233 PMC4043367

[ref49] Nizamutdinov D. Qi X. Berman M. H. Dougal G. Dayawansa S. Wu E.. (2021). Transcranial near infrared light stimulations improve cognition in patients with dementia. Aging Dis. 12, 954–963. doi: 10.14336/AD.2021.0229, PMID: 34221541 PMC8219492

[ref50] Oron A. Oron U. Streeter J. De Taboada L. Alexandrovich A. Trembovler V.. (2007). Low-level laser therapy applied transcranially to mice following traumatic brain injury significantly reduces long-term neurological deficits. J. Neurotrauma 24, 651–656. doi: 10.1089/neu.2006.0198, PMID: 17439348

[ref51] Pantano P. Baron J. C. Lebrun-Grandié P. Duquesnoy N. Bousser M. G. Comar D. (1984). Regional cerebral blood flow and oxygen consumption in human aging. Stroke 15, 635–641. doi: 10.1161/01.STR.15.4.635 6611613

[ref52] Pfeffer R. I. Kurosaki T. T. Harrah C. H. Chance J. M. Filos S. (1982). Measurement of functional activities in older adults in the community. J. Gerontol. 37, 323–329. doi: 10.1093/geronj/37.3.323 7069156

[ref53] Pfefferbaum A. Adalsteinsson E. Sullivan E. V. (2005). Frontal circuitry degradation marks healthy adult aging: evidence from diffusion tensor imaging. NeuroImage 26, 891–899. doi: 10.1016/j.neuroimage.2005.02.034, PMID: 15955499

[ref54] Poyton R. O. Ball K. A. (2011). Therapeutic photobiomodulation: nitric oxide and a novel function of mitochondrial cytochrome c oxidase. Discov. Med. 11, 154–159. 21356170

[ref55] Qi X. Nizamutdinov D. Berman M. H. Dougal G. Chazot P. L. Wu E.. (2021). Gender differences of dementia in response to intensive self-administered transcranial and intraocular near-infrared stimulation. Cureus 13:e16188. doi: 10.7759/cureus.16188, PMID: 34262831 PMC8260213

[ref56] Raz N. Gunning F. M. Head D. Dupuis J. H. McQuain J. Briggs S. D.. (1997). Selective aging of the human cerebral cortex observed in vivo: differential vulnerability of the prefrontal gray matter. Cereb. Cortex 7, 268–282. doi: 10.1093/cercor/7.3.268, PMID: 9143446

[ref57] Raz N. Lindenberger U. Rodrigue K. M. Kennedy K. M. Head D. Williamson A.. (2005). Regional brain changes in aging healthy adults: general trends, individual differences and modifiers. Cereb. Cortex 15, 1676–1689. doi: 10.1093/cercor/bhi044, PMID: 15703252

[ref58] Salat D. H. Tuch D. S. Greve D. N. van der Kouwe A. J. W. Hevelone N. D. Zaleta A. K.. (2005). Age-related alterations in white matter microstructure measured by diffusion tensor imaging. Neurobiol. Aging 26, 1215–1227. doi: 10.1016/j.neurobiolaging.2004.09.017 15917106

[ref59] Salehpour F. Hamblin M. R. DiDuro J. O. (2019). Rapid reversal of cognitive decline, olfactory dysfunction, and quality of life using multi-modality photobiomodulation therapy: case report. Photobiomodul. Photomed. Laser Surg. 37, 159–167. doi: 10.1089/photob.2018.4569, PMID: 31050946

[ref60] Salgado A. S. Zângaro R. A. Parreira R. B. Kerppers I. I. (2015). The effects of transcranial LED therapy (TCLT) on cerebral blood flow in the elderly women. Lasers Med. Sci. 30, 339–346. doi: 10.1007/s10103-014-1669-2, PMID: 25277249

[ref61] Salthouse T. A. (2010). Selective review of cognitive aging. J. Int. Neuropsychol. Soc. 16, 754–760. doi: 10.1017/S1355617710000706, PMID: 20673381 PMC3637655

[ref62] Sharma S. K. Kharkwal G. B. Sajo M. Huang Y. De Taboada L. McCarthy T.. (2011). Dose response effects of 810 nm laser light on mouse primary cortical neurons. Lasers Surg. Med. 43, 851–859. doi: 10.1002/lsm.21100, PMID: 21956634 PMC3199299

[ref63] Sheppard F. R. Kelher M. R. Moore E. E. McLaughlin N. J. Banerjee A. Silliman C. C. (2005). Structural organization of the neutrophil NADPH oxidase: phosphorylation and translocation during priming and activation. J. Leukoc. Biol. 78, 1025–1042. doi: 10.1189/jlb.0804442, PMID: 16204621

[ref64] Spreng R. N. Wojtowicz M. Grady C. L. (2010). Reliable differences in brain activity between young and old adults: a quantitative meta-analysis across multiple cognitive domains. Neurosci. Biobehav. Rev. 34, 1178–1194. doi: 10.1016/j.neubiorev.2010.01.009, PMID: 20109489

[ref65] Tafur J. Mills P. J. (2008). Low-intensity light therapy: exploring the role of redox mechanisms. Photomed. Laser Surg. 26, 323–328. doi: 10.1089/pho.2007.2184, PMID: 18665762 PMC2996814

[ref66] Uozumi Y. Nawashiro H. Sato S. Kawauchi S. Shima K. Kikuchi M. (2010). Targeted increase in cerebral blood flow by transcranial near-infrared laser irradiation. Lasers Surg. Med. 42, 566–576. doi: 10.1002/lsm.20938, PMID: 20662034

[ref67] Van der Elst W. Van Boxtel M. P. Van Breukelen G. J. Jolles J. (2006). Normative data for the animal, profession and letter M naming verbal fluency tests for Dutch speaking participants and the effects of age, education, and sex. J. Int. Neuropsychol. Soc. 12, 80–89. doi: 10.1017/S1355617706060115, PMID: 16433947

[ref68] Vargas E. Barrett D. W. Saucedo C. L. Huang L. Abraham J. A. Tanaka H.. (2017). Beneficial neurocognitive effects of transcranial laser in older adults. Lasers Med. Sci. 32, 1153–1162. doi: 10.1007/s10103-017-2221-y, PMID: 28466195 PMC6802936

[ref69] Villringer A. Chance B. (1997). Non-invasive optical spectroscopy and imaging of human brain function. Trends Neurosci. 20, 435–442. doi: 10.1016/S0166-2236(97)01132-6 9347608

[ref70] West R. L. (1996). An application of prefrontal cortex function theory to cognitive aging. Psychol. Bull. 120, 272–292. doi: 10.1037/0033-2909.120.2.272, PMID: 8831298

[ref71] West R. Alain C. (2000). Age-related decline in inhibitory control contributes to the increased Stroop effect observed in older adults. Psychophysiology 37, 179–189. doi: 10.1111/1469-8986.3720179, PMID: 10731768

[ref72] Wong M. T. P. Ho T. P. Ho M. Y. Yu C. S. Wong Y. H. Lee S. Y. (2002). Development and inter-rater reliability of a standardized verbal instruction manual for the Chinese geriatric depression scale—short form. Int. J. Geriatr. Psychiatry 17, 459–463. doi: 10.1002/gps.633, PMID: 11994935

[ref73] Wong-Riley M. T. Bai X. Buchmann E. Whelan H. T. (2001). Light-emitting diode treatment reverses the effect of TTX on cytochrome oxidase in neurons. Neuroreport 12, 3033–3037. doi: 10.1097/00001756-200110080-00011, PMID: 11568632

[ref74] Wong-Riley M. T. Liang H. L. Eells J. T. Chance B. Henry M. M. Buchmann E.. (2005). Photobiomodulation directly benefits primary neurons functionally inactivated by toxins: role of cytochrome c oxidase. J. Biol. Chem. 280, 4761–4771. doi: 10.1074/jbc.M409650200, PMID: 15557336

[ref75] Ying R. Liang H. L. Whelan H. T. Eells J. T. Wong-Riley M. T. (2008). Pretreatment with near-infrared light via light-emitting diode provides added benefit against rotenone and MPP-induced neurotoxicity. Brain Res. 1243, 167–173. doi: 10.1016/j.brainres.2008.09.057, PMID: 18848925 PMC3706077

